# Foreign Medico-Psychological Literature

**Published:** 1862-10

**Authors:** 


### FOREIGN MEDICO-PSYCHOLOGICAL LITERATURE.

OUR Retrospect of current Foreign Medico-Psychological Literature will embrace the following subjects:

1. The Relations of Inebriety to Insanity.
2. Cases of Fracture of the Ribs in Insane Patients discovered after Death.
3. On Moral Insanity in Relation to Criminal Acts.
4. The Diagnosis of certain Forms of Insanity.
5. The Influence of the Civil War in America upon Insanity.

1.—*The Relations of Inebriety to Insanity.* By GEORGE COOK, M.D., Brigham Hall, Canandaigua, N. Y.

WE extract the following paragraphs from an Essay under this title published in the *American Journal of Insanity* (April, 1862):—

"There is a class of young inebriates found in asylums. Some of these have never known and felt the wholesome restraining influences of home life; others, having early thrown off the parental control, are quickly developed by vice and its associations into 'fast boys,' reckless alike of the past and future. They become passionate, profane, licentious, drunken; without any guiding and controlling motives, they are useless and dangerous, and as a last hope their friends send them to an asylum.

"Another class is composed mostly of men, who day by day drink to excess, or have periods of debauch, with more or less frequency and regularity. These last are the paroxysmal dipsomaniacs. The habit, in these cases, is usually confirmed by many years of indulgence, and the brain and nervous system not unfrequently suffer in consequence. Their power of self-control is weakened, perhaps overcome under temptation, by the master force of habit, or they are in a state of chronic alcoholism. Some of these men lament their condition, strive to regain the mastery over their appetite for liquor, and voluntarily yield themselves to seclusion and restraint.

"A third class of cases, small in number, come under the care of the medical officers of asylums. The origin of the habit dates back to the days of boyhood, but as years pass, and these men engage in the active cares and duties of life, the habit is for a time suspended, or indulged in secret; occasionally they break away from the restraints imposed by the will or by worldly considerations, and are suddenly, without reflection, hurried into excess and the gutter; from thence to the wards of a hospital or lunatic asylum.

"A fourth class is composed of females addicted to the use of opium and alcohol.

"Cases of mingled insanity and intemperance are common. They may be found reported under the forms of mania, general and partial, melancholia, and general paralysis. But if there are cases of inebriety, or dipsomania, as described by Dr. Hutcheson, received into American asylums which do not belong to one of the foregoing classes, it has not been my fortune, in a period of observation extending over thirteen years, to meet with them. Perhaps others, with more extended opportunities for observation may have seen other phases of this vice. Judging, however, from the reports of the medical superintendents of American asylums, dipsomania must be exceedingly rare among our people. Dr. Stokes, of the Mount Hope Hospital, Baltimore, reports sixteen cases in 1859, and Dr. Tyler, of the McLean Asylum, Boston, in his report for the same year, mentions the occurrence of such a form of disease. With these two exceptions, I have looked in vain for any reported cases in American asylums, for the years 1858–9. Dr. Skae, of the Royal Edinburgh Asylum, treated four cases in 1859, and, during the same year, there were four cases in one thousand seventy-two patients received at the Bicêtre and Salpêtrière, all of them females.

"In view of all the facts recorded and observed upon this question, I can only conclude, that intemperance is entitled to the same place among the symptoms of insanity as any other human appetite or action; that when associated with other symptoms of cerebral disorder, it may, and often does, indicate insanity; that when unaccompanied by such symptoms, it ought not to be regarded as a symptom even—much less should it be considered as forming a specific type—of mental disease. Hence, I believe the name applied to it to be not only 'a needless refinement,' but incorrect, and productive of evil consequences. Dipsomania means, if it means anything, that the patient is in a state of mania, specific in form, and having a peculiar and specific pathology, indicated by a craving for alcohol so strong and imperious as to overpower the will by its morbid influence. And we are told by some observers that the thirst for alcohol alone is sufficient evidence of insanity, upon which to rest an opinion; that it is pathognomonic! We are further informed that all those who venture to reach any other conclusion than this, are departing from the orthodox ways of psychology, and calling in question the existence of a form of mental disease recognised by Esquirol and 'considered as well established as any other.' I have given Esquirol's opinions, and some of his cases, and have shown that he did not distinctly recognise, as a form of disease, this chronic and paroxysmal drunkenness; and I submit that a study of cases does not warrant such assumptions as those stated above. * * *

"Intemperance is frequently a concomitant of mental derangement. Among the patients received into our asylums are many whose previous habits have been, more or less, irregular and intemperate. The mental disease is traceable to other causes than alcoholic excesses, but as the healthy cerebral action gives place to the morbid, the healthy balance of the functions is impaired, the propensities assume a more controlling influence, and the appetites crave a more frequent indulgence. In these, intemperance is only an exaggeration of a habit or propensity; it holds neither the relation of cause or symptom to the mental disease, but is simply one of its concomitants. No particular consideration or examination of these cases is necessary; I thus briefly allude to them as showing one of the relations of inebriety to insanity.

"The power of habit, as it influences and controls human thoughts and actions, is seen and felt by all men. The parent bears in mind this potent influence, and endeavours to 'train up his child in the way he should go;' the educator makes it subserve his purpose, and the moralist and physician have to do with its good and evil effects, in forms without number. In many individuals, the power of habit seems to bear imperial sway, and the will either bends its utmost energies in aid of the usurper, or feebly struggles to free itself. Few men possess such organizations as never to feel its influence. In most men, actions, whether good or bad, tend to repeat themselves, and when frequently practised they acquire a power of reproduction independent of volition. I have known a vicious habit formed in early boyhood, suspended by a change of circumstances, and again resumed, later in life, on a return to the early associations and opportunities. And when associated with the appetites and propensities, who shall measure accurately the extent of this power of habit? Is it necessary to conclude, therefore, that every man is insane, who either does not, or cannot, control his appetites? This irresistible impulse for alcohol, which is considered as forming the essential nature of paroxysmal and chronic dipsomania, is it anything more than the power of habit and alcoholism? We see the same impelling force, the same binding of the human will to the yoke of servitude, the same resulting degradation of mind and body, in the opium-eaters of the East. Undoubtedly the nervous system is disturbed, and the brain suffers under the use of the narcotic, but are we prepared to make opium-eating, when the habit assumes a controlling power, and the appetite demands indulgence, a special form of insanity? Haschish has its millions of slaves; are they too forced to use this potent drug by the influence of primary cerebral disease?

"We are told by the very highest metaphysical authority, that 'as a man soweth, that shall he also reap,' and every man's life illustrates the truth of these words. A man voluntarily yields himself to some demoralizing indulgence for the first time, and a breach once made in the walls of self-respect and self-control, a second indulgence meets with less resistance, a third with still less; each repetition of the act gradually acquiring a power of its own, until, by frequent indulgence, a myriad of broken resolutions lie buried in indifference and despair, and habit sways the sceptre of dethroned manhood. The seeds of intemperance are generally sown with some care. No frequent or excessive indulgence is intended, but time passes, and the master who either cautiously or heedlessly scattered the seeds of vice, becomes thereby transformed into the slave, reaping a bitter harvest of weakness and drunkenness.

"I believe I do not err when I say that this all-pervading element of human character, in its relations to the special phases of inebriety, has not received the attention it merits. Some observers seem to ignore its existence, and hastily assume that a large class of confirmed inebriates are suffering under mania; others, while recognising the force and power of habit, and the effects of alcohol, suffer themselves to depart from the very principles of diagnosis which they profess to follow. Thus it happens that the habit of drunkenness is too often considered by medical men as forming a type of insanity, under the specious term of dipsomania. * * *

"I conclude from all the facts and observations within my reach—

"1st. That inebriety or intemperance frequently holds the relation of cause to mental disease. This effect of alcohol was observed and recorded by the earliest writers upon insanity, and the evidence of its increasing influence may be found in the subsequent observations of all writers upon the causes of mental derangement, and in modern hospital statistics.

"2nd. Intemperance is entitled to a place among the symptoms of insanity when it is preceded or accompanied by other evidences of disease of the nervous centres. It should be borne in mind, in considering this relation of inebriety to insanity, that we have to look, not alone upon the series of acts called intemperance, but also upon the cause of these acts; for there is no single human action, taken apart from the mental condition from which it springs, from the previous character and habits, and the surrounding circumstances, which indicates beyond all question the existence of insanity. When the act is clearly prompted by delusion, the question is relieved of all difficulty, but when the intellectual disturbance is not apparent, and only uncontrollable desire or irresistible impulse inferred from the very act which we are called to examine, in its relation of symptom to certain pathological changes, then we must have something more than the act, we must have evidence that the act is the result of disease, we must have the ordinary symptoms indicating the existence of the pathological changes, or else we have no legitimate authority for the conclusion that it proves the existence of insanity. And such a conclusion has no other weight than is given it by our own individual opinion. When evidence of disease of the brain and nervous system is manifest, the act, the outgrowth of the disease, takes its proper place among the symptoms. It does not become the disease, or even the essential character of the disease, nor ought the nosological place of the malady to be determined thereby.

"I further conclude that the physiological power of habit, acting at the moment of temptation, displacing or overruling the action of the will by its impetuous force or unceasing craving, is the foundation on which rests the superstructure of inebriety, in all its chronic and paroxysmal forms. I am not now speaking of the effects of the alcoholic poison described by various writers under the term alcoholism, or of those exceptional cases, of comparatively rare occurrence, in which intemperance is suddenly developed with other marked changes of habit, character, and physical condition, but of the large class of inebriates from volition and habit, around whom many members of the medical profession are disposed to throw the mantle of insanity and irresponsibility. While I would extend to this unfortunate class of persons the utmost sympathy and assistance, and afford to them the restraining and reformatory influences of an asylum, I would urge the importance of carefully discriminating between them and the sufferers under cerebral disease. The medical, moral, and legal relations of each class demand this at our hands.

"There remains the small number of cases in which alcoholic craving and indulgence, with more or less prominence, co-exist with other unmistakeable symptoms of cerebral disease. From such cases comes the name dipsomania, which I regard as erroneous and mischievous. Bequeathed to us by early writers and authorities upon insanity, this term has been made, by subsequent writers, to cover the habit of intemperance as manifested in its chronic and paroxysmal forms, mingled as it advances with the phenomena of alcoholism." —*American Journal of Insanity*, April, 1862.

---

2.—*Cases of Fracture of the Ribs of Insane Patients, revealed by Post-Mortem Examination.* By Joseph Workman, M.D.

"J. A., aged 33, reported before admission to be a most furious and dangerous lunatic. On admission he was pale, as if from inanition and want of sleep. He was restless, noisy and destructive at first, but in the course of three weeks he became quiet and harmless, took his food well, and appeared to rest well at night. He complained of no pain whatever, and had no cough. On the thirty-third day after admission, I observed œdema of the feet, and ordered that he should go to bed, prescribing such remedies for the dropsical symptoms as were thought proper. Next day the œdema had extended to the legs, and it continued to increase. On the third day, clear indications of hydrothorax were observable, but from his restlessness close examination was impossible. He died on the fourth day.

"The post-mortem showed the left thorax completely filled with water, the right thorax half full, and about three ounces in the pericardium. The abdomen also presented dropsical effusion.

"Seven ribs were found fractured, and presented very imperfect marks of restorative action. The condition of the broken ends, and the whole appearance of adjacent parts, proved satisfactorily that the fractures were of a date more remote than that of his admission; and I felt convinced that they had taken place in the gaol from which he was sent, or more probably at home, before his commitment. The brain presented a highly congested condition, and other marks of inflammatory action were seen. The lateral ventricles contained about an ounce and a half of serum.

"D. C., a tall and powerful-looking man, admitted 17th December, 1861, and certified to have been insane for only 8 weeks previously. He presented, on entrance, unmistakeable indications of general paralysis. He was very noisy, and spoke with great authority; in fact, he was hardly ever silent, and though he spoke with the peculiar lingual drag of patients of his class, there was no want of volume in his voice. He said he had no pain; his appetite was very keen; and I marked him down as an early victim to general paralysis. He continued to go about until six days before his death, when an apparent aggravation of his paralytic impairment presented, and he was confined to his bed. He gradually became more feeble, but had no coma. He could swallow, though with difficulty, until a few hours before his death, which took place on the forty-ninth day from his admission.

"So little suspicion had I of the existence of any other disease than that of the brain, that on the completion of this portion of the autopsy I left the dead-room, saying to my assistants, they need not trouble themselves with any further search, as the patient's abdominal and thoracic organs were doubtless quite sound. But they were not satisfied to relinquish their labours. They proceeded to the opening of the thorax and abdomen, and, on dissecting back the integuments preparatory to cutting through the ribs, they detected, on the right side, beneath the *pectoralis major*, deposits of dark pus at two points, which proved subsequently to be seats of fracture. The 1st, 2nd, 3rd and 4th ribs were found fractured, about an inch from the cartilaginous ends. The cyst of the upper deposit of pus was over the fracture of the first rib; the lower one extended over the other three fractures. Under the microscope scarcely a single pus-globule was discernible; so that the deposit could not have been recent. No separation had taken place. The right pleura was adherent to the fourth rib, and to the space between the second and third ribs. The fractures ranged in a straight line, as if all caused by one blow, or most probably by a fall on some hard-edged substance. In neither side of the thorax was there any deposit of serum worthy of notice; and the lungs were both healthy.

"The pericardium contained about three ounces of serum, and the heart presented partial fatty degeneration.

"The scalp showed an old cicatrix, about an inch and a half from, and behind the anterior fontanelle. The dura mater was adherent to the skull from the anterior fontanelle backward over the whole summit; and it was adherent to the brain from the same point backward, along the great fissure, about one and one-fourth inches on each side. A considerable quantity of fluid was diffused over the whole brain, beneath the pia mater. The meningeal vessels were considerably congested, but slices of the brain, under the microscope, showed little vascularity. There was general œdema of the brain substance, and it had this form of softening only. On the base of the brain fully three ounces of serum were found; and behind the tentorium about one ounce.—*American Journal of Insanity*, April, 1862.

---

### 3.—*On Moral Insanity in Relation to Criminal Acts.* By Dr. J. Parigot.

The following extracts are from a paper read by Dr. Parigot before the New York Academy of Medicine:—

"Recent analysis of the functions of the mind has shown that its several operations can be divided into five faculties: 1st. Sensation. 2nd. Moral feeling or emotion (both of the receptive order). 3rd. Intellectual power. 4th. Volition (the only faculty according to the signification of the word); and 5th. The natural instincts. The three last belong to human activity; it has been found, also, that any trouble, exaltation, depression, abolition, or perversion of any of our mental functions, when accompanied (as always is the case, more or less evidently,) with physical symptoms, was sufficient as good evidence before courts of justice. In fact, the study of psychologico-forensic medicine and its progress are, in a certain measure, the result of several cases of moral insanity which attracted great notoriety. The perusal of those trials is of the greatest importance for our studies. It may be seen that a great number of the accused were in a very extraordinary mental condition; the *unity* of their mind being in a certain measure destroyed, since they were in a struggle, trying to collect their ideas and feelings in order to master wild impulses! In almost each trial, in which moral insanity is the plea of defence, the prosecution maintains that such a disease does not exist, and brings forth examples and books to sustain this assertion: lawyers not being able to distinguish the disease by its morbid symptoms, pretend that criminals are more or less *morally insane*—*id est*, wicked, dissolute, and perverted. On the other side, the defence has often resorted to this plea of insanity as a remaining chance of acquittal; some physicians, moved by a desire to wrest from the scaffold some prisoners that appeared to them more deprived of reason than malicious and wicked, have gone too far in their so-called philanthropic endeavour.

"In spite of many discussions, held in medical societies and academies, doubts concerning the theory of moral insanity, for legal purposes, have not yet been resolved. The reason of it may lie in the fact, that if people consider moral insanity from the point of view of its flagrant attacks and of its terrible results on society; if, at the same time, the criterion of knowing right from wrong has been employed as a test, then the logical inference is, that such acts must be repressed and their perpetrators punished. But, on the other side, if physical and psychical symptoms agree in demonstrating a disease of the brain, then it must be evident to courts, juries, and lawyers, that the offender, at the moment he committed crime, had no power to control his will, nor to choose right from wrong; that he was insane, because he could not dominate a morbid impulse, or that he was not able to adequate his actions to a real motive. * * *

"Now, Esquirol said that the difference to be found in a mad-house and the world was only in a more accentuated shade of mad ideas, errors, and passions or propensities; metaphysical science finds also no line that separates reason from madness. The celebrated Lelut—member of the French Institute—says, in a memoir on insanity, 'that in its beginning, insanity is still reason, as reason is already madness! That mental predisposition, which may be organical cause of insanity, consists (for the *moral or sentimental sphere*) in excessive irritability and sensibility; then appear strange desires, *perverted inclinations* and tendencies, bad passions, &c.; for the *intellectual sphere*, they consist in want of attention, which leads to absence of mind, giving to the person an appearance of insensibility to external impressions; then a vicious association of feelings; ideas produce irregularity and discrepancies in words and phrases; or a too rapid association of ideas brings on confusion of speech, incoherence and unintelligible ellipses of thought; at last the symptoms of madness show themselves in false judgments, leading to wrong opinions, *determinations and acts* opposed to social order and morality.' Well, if there is no psychical demarcation between reason and madness, why not have recourse to the physical one? It is admitted that it is necessary to compare the actual state of the individual to his previous state of mind and body, in order to appreciate the quantity or degree of existing differences; but if that person was a little eccentric, would not serious difficulties arise, unless physical symptoms could be ascertained. Every one may understand that, under such circumstances, lawyers tried to exclude physicians when cases could not be easily ascertained. 'What,' said an attorney for the Crown, 'a so-called monomaniac pleads guilty; he knows what he has done; he was aware of the penalty that his crime deserves; he knows even the law which forbids such an action; and now medical men come here pretending that such a man is not guilty!' The answer is this;. If it is proved by the history of the case that there was no adequate motive; that the perpetrator of the crime was not in possession of his free will; that an impulse forced him to the action; if anamnestic evidence is in his favour; if physical signs of insanity do exist—we say, that man being of unsound mind, no penal law can be applied to him; but it is your right to employ any means, consonant with civilization, that you judge to be the best to prevent any future accidents or hurt to anybody from his disease. * * *

"Now passion, hatred, anger, animal and selfish inclinations, &c., do not in the least destroy our liberty and volition. Instantaneous madness may happen, but it is very rare; in these cases latent symptoms have not been observed—that is all. Decision has always, in one form or another, preceded action, and our conscience has approved or rejected our motives; this is so true, that criminals have confessed to have been obliged to get themselves under the influence of liquor, to be enabled to carry out their plans. In this case drunkenness is no more an excuse than passion could be; and will never be considered as an excuse, because free-will was purposely diminished or oppressed!

"In insanity free-will no longer exists, on account of a material condition of the mind, and therefore good sense requires that such a condition should be ascertained medically. * * *

"In our estimation, Diastrephia [a term for volitional insanity which Dr. Parigot would introduce], has the same relation to an act that delusion has to a thought; they are two equal terms, indicating the error and guile of an insane person; but the first is much more important and prejudicial than the second, which concerns only our individuality. There is an obvious reason why the terms of such a sort of algebraic equation cannot be inverted; it is, that logic and grammar do not permit to express the delusion or delirium of an act meaning its folly or insanity. Diastrephia is a special perversion, only applicable to volition and instincts. By this distinction authors on pathology are able to classify that sort of infirmity.

"From this point of view, insanity, considered in its true objective relation, furnishes us with a definition that meets better any form of insanity for forensic practice. It is no more to be said to be a total or partial deprivation of the power of reasoning and of distinguishing right from wrong, nor is the general character of insanity any longer an emotional trouble, as the celebrated Guislain called it; neither can it be said to be a disease of our perceptive faculties, with subsequent loss of judgment. All these phenomena are characteristics of certain orders of disease, but not applicable to all cases, and especially to those requiring forensic discussion. For law purposes, insanity might be defined *the loss of power of control either over one or more of our mental faculties, including especially the absence of free-will, demonstrated by moral and physiological symptoms.*

"In a medical point of view, it is *an idiopathic or sympathetic disease of the brain, which interferes with the psychological and physiological functions of this organ.* From the stand point of administrative authority that has charge of preserving the peace and security of cities, towns, or villages, *insanity begins only when a patient endangers the community or his own life and property.* * * * *

"Now, supposing a judicial case in which immorality should have been the efficient cause of a mental disease, vice should have taken a morbid existence; the prosecution says that it is clear that the man is immoral, and is only trying to escape punishment; public opinion is against the plea of insanity, as being fallacious: here the physician will be the only one unprejudiced, and in spite of all influence, relying only on the unequivocal signs of material disease in connexion with psychical symptoms, he settles the case to the satisfaction only of his conscience. Sudden violent and ungovernable passions are not symptomatic of diastrephia—*nemo repente furiosus;* it requires a certain time before passions or habits can inflict on us either a derangement of functions or a change in our tissues; the power, also, of unbridled organic propensities is of long and gradual effect on our mind. * *

"We say, if pathological symptoms cannot be traced *clearly*, if psychological symptoms are *doubtful*, a medical man cannot give his evidence in favour of insanity." * * *

The paper concludes with the following propositions:—

"I. That the disease called moral insanity is but an affection of the faculty of volition and instincts, always attended by physical and physiological symptoms.

"II. That the name of moral insanity is defective, because it bears no relation to its cause, symptoms, and results; and that it misleads the opinions of the bar concerning crimes committed under its influence.

"III. That the laws and rules concerning insanity, relating to civil and criminal cases, ought to be made conformable to the actual state of medical science.

"IV. That no person ought to be considered as being insane, if physical and mental signs cannot be traced and ascertained.

"V. That a reform concerning medical certificates is necessary, to insure regularity in obtaining from courts or judges orders to detain a person as being insane; that no such document be admitted, unless containing,

"1st. All the anamnestic, physical, physiological, and mental symptoms of the case.

2nd. The diagnosis and prognosis of the disease."—*American Journal of Insanity*, April, 1862.

---

4.—*On the Diagnosis of Certain Forms of Insanity.* By Dr. Curwen.

"No definition of insanity has ever yet been given which would clearly and distinctly include all the variations to be met with. Certain forms may be very readily recognised and very clearly described, but there is still a large class of mental disorders which defy all accurate definition, and are a source of grievous perplexity to all who have not made them their special study. In this class are included all those cases of mental disorder which have proved such obstacles to judges, lawyers, and juries, and have even caused much division of opinion among medical men.

"To arrive at correct conclusions relative to this latter class, it does not become us to set up some imaginary standard, clothed with certain characteristics which we conceive should belong to it, but we should carefully and judiciously compare the conduct, character, the expressions and general tone and peculiarities of the conversation, with what the individual was before the character we are now examining was developed; for we must constantly bear in mind that insanity is not some new thing engrafted on the individual, but in all cases is a perversion of his mental and moral powers, made known by the change in his demeanour, his affections, his passions, and all those peculiar characteristics which go to make up the individual man.

"It may not be possible in this way to come at once to a conclusion in all cases, but justice to the individual and the cause of truth demands that such a course should be rigorously pursued. There may be, as there have heretofore been, cases which for a time will apparently fail to be decided by this criterion, but if the investigation is pursued cautiously, prudently, and in the spirit of an earnest seeker after truth, the result cannot be long doubtful.

"By this criterion we shall be able to decide those cases also for which medical men, or more strictly, physicians for the insane, have been said to have invented a term which should serve as a cloak for crime, and prevent the execution of the penalty of the law on men who have committed gross violations of the rights of their fellows.

"That such a doctrine has been used by ingenious advocates to attempt to screen great offenders cannot be denied; but because such doctrines have been abused by those who were more anxious to serve their client than to advance the cause of truth and justice, only proves that the best objects may be perverted to the worst purposes.

"The statement is not that men who have led a vicious life until their consciences are seared as with a hot iron, are morally insane, but that men who have led correct and upright lives, have been all that could be desired as members of society, have, after an attack of fever, or some severe bodily disease, had their moral powers so perverted as to lead them to the commission of acts at which formerly they would have revolted, and their mental powers so weakened as to prevent the perception of the incongruity of their actions with their professions." —(*Report of State Lunatic Hospital of Pennsylvania*, 1861; *American Journal of Insanity*, April, 1862.)

---

5.—*The Influence of the Civil War in America on Insanity.* By Dr. Stokes.

"In most of the cases occurring since the 19th of April, '*excitement of the times*' has entered more or less directly into the causation of every attack. Our experience therefore fully corroborates the remark of Pinel, that it would not be difficult to retrace, among the admissions into our insane establishments, the form of insanity especially appropriate to the exaltation of ideas prevalent at each epoch. One is the victim of the monomania of fear, and entertains the delusion that he is suspected of being a spy. He passes his days and nights in constant apprehension of being arrested and imprisoned. Fearing to go to sleep, he lies awake listening to every noise, and as his ear catches the sound of the distant footstep, he becomes dreadfully terrified and alarmed. His morbid and affrighted imagination conjures up before him the dreaded spectre of an officer intent upon his arrest. He begs to be permitted to deliver himself up to the authorities, and yet trembles when the desired opportunity is promised him. Another entertains the belief that he is suspected of disloyalty to the Government, and imagines that the eyes of every passing stranger are specially directed upon him—noticing and watching his every word and action. Dreading the confiscation of his property, he assigns over all he possesses to his friend. Then, to escape the dreaded disgrace of imprisonment, he eludes the vigilance of his family, and in a short time is brought back, having made an unsuccessful attempt to drown himself. A third loses his mind in consequence of the startling events of the 19th of April. He conceives the idea that he is believed to have taken an active part in the movement of that day, and to escape arrest, imprisonment, and execution, he makes a desperate attempt at suicide. With a razor he makes a ghastly gash in his throat, severing the trachea, but missing the carotids. For a whole month after this, this terrible fear overshadows his mind, and the suicidal propensity abates nothing of its determined character. Three several times he tears the wound asunder, by the violent moving and twisting of his head, though his hands are confined in the muff, and he secured in his bed. By keeping his head firmly planted and nestled in an apparatus well wadded and stuffed all around, and by having an attendant beside him night and day, the wound is maintained in apposition and finally perfectly healed. It united, leaving scarce a scar, and the patient fully recovered his mind. A fourth is the subject of aural delusions. As soon as he drops off to sleep at night, he is roused from his slumbers by awful sounds in the far distance. Night after night he passes in intently listening to the terrible clash of armies in deadly strife. He imagines he hears the distant roar of artillery, the heavy tramp of thousands of horses, and the deadly rush of cavalry. Then reach his ear, with frightful vividness and distinctness, the moans and groans of the wounded and dying, the shrieks of captive women and their cries of anguish and horror."—*Report of Mount Hope Institution, near Baltimore. American Journal of Insanity*, April, 1862.

